# Cancer-associated fibroblast–secreted collagen is associated with immune inhibitor receptor LAIR1 in gliomas

**DOI:** 10.1172/JCI176613

**Published:** 2024-02-15

**Authors:** Shashwat Tripathi, Hinda Najem, Corey Dussold, Sebastian Pacheco, Jason Miska, Kathleen McCortney, Alicia Steffens, Jordain Walshon, Daniel Winkowski, Michael Cloney, Matthew Ordon, William Gibson, Hanna Kemeny, Mark Youngblood, Rebecca Du, James Mossner, Pavlos Texakalidis, Annelise Sprau, Matthew Tate, Charles David James, Craig M. Horbinski, Nitin R. Wadhwani, Maciej S. Lesniak, Sandi Lam, Ankita Sati, Manish Aghi, Michael DeCuypere, Amy B. Heimberger

**Affiliations:** 1 Department of Neurological Surgery,; 2 Malnati Brain Tumor Institute, Robert H. Lurie Comprehensive Cancer Center, and; 3 Department of Pathology, Feinberg School of Medicine, Northwestern University, Chicago, Illinois, USA.; 4 Visiopharm, Horsholm, Denmark.; 5 Department of Pathology & Laboratory Medicine and; 6 Division of Pediatric Neurosurgery, Ann & Robert H. Lurie Children’s Hospital of Chicago, Chicago, Illinois, USA.; 7 Department of Neurological Surgery, UCSF, San Francisco, California, USA.

**Keywords:** Cell Biology, Oncology, Brain cancer, Collagens, Macrophages

**To the Editor:** A recently published *JCI* paper revealed that cancer-associated fibroblasts (CAFs) are present in glioblastoma and are defined by the presence of 9 transcriptional markers (1). CAFs can form a barrier around cancer cells that prevents their detection by T cells (2). The normal brain has low levels of collagen, but deposition increases in gliomas, especially around vessels. In addition to collagen facilitating tumor invasion and providing a niche for cancer stem cells, collagen has also been shown to trigger immune suppression through LAIR1-mediated T cell exhaustion and alternative activation of macrophages (3, 4).

CAFs were identified from single cells isolated from 21 samples (high-grade gliomas [HGGs], 5; pilocytic astrocytomas [PAs], 13; and normal brains [NBs], 3) by examining differential expressed genes against known CAF markers and CAF subtype markers (Figure 1A). The percentage of CAFs was a function of glioma grade, with 1% CAFs present in NBs/low-grade gliomas and nearly 20% in HGGs with an enrichment of the immunomodulatory CAF subtype (Figure 1, B and C). Gene ontology analysis suggested CAF-mediated immune suppression and migration inhibition (Figure 1D).

To ascertain the collagen types produced by CAFs, scRNA-Seq data from our pediatric glioma cohort complemented with public adult glioblastoma data sets (5) were analyzed and showed that CAFs expressed *COL6A1/A2* > *COL4A1*, *COL9A3*, *COL12A1* > *COL1A* (Figure 1E and Supplemental Figure 1A; supplemental material available online with this article; https://doi.org/10.1172/JCI176613DS1). ELISA confirmed collagen production by human glioblastoma CAFs at 773 μg/mL. Within the public data set (5), the CAF populations are included in the pericyte compartment. Pericytes expressed the greatest diversity of collagen subtypes and were the main source of *COL1A1/A2*, *COL3A1*, *COL4A1/A2*, *COL5A1/A2*, *COL6A1/A2/A3*, and *COL18A1*; endothelial cells were also a source of *COL4A1/A2* and *COL18A1*. Glioma cells expressed modest levels of *COL6A1* and *COL9A3* (Supplemental Figure 1A). Prognostic analysis of collagen subtypes from The Cancer Genome Atlas revealed that 6 types were negatively associated with outcome, including *COL1A2* (high vs. low expression, 12.3 vs. 16.1 months; HR = 0.68; *P* = 0.03); *COL6A1* (11.9 vs. 15.9 months; HR = 0.53; *P* = <0.01); *COL8A2* (12.6 vs. 15 months; HR = 0.65; *P* = 0.02); *COL22A1* (11.8 vs. 15.4 months; HR = 0.67; *P* = 0.03); *COL24A1* (11.8 vs. 15 months; HR = 0.64; *P* = 0.01); and *COL27A1* (11.8 vs. 15.8 months; HR = 0.63; *P* = 0.01) (Supplemental Figure 1B). Among the most frequently expressed and prognostic collagens, *COL1A2*, *COL4A1*, and *COL6A1* were enriched near the hyperplastic vessels and microvascular proliferation, whereas *COL1A2* and *COL4A1* were enriched in the perinecrotic regions (Supplemental Figure 1C). To validate these findings, multiplex imaging demonstrated that CAFs were embedded in COL6A1/A2 within HGGs (Figure 1F and Supplemental Video 1). COL1A and COL4A were expressed in tumor glomeruloid structures (Supplemental Figure 2A). ACTA2^+^ arteries, with COL4A expressed in the vasculature wall, were embedded in a COL1A^+^ matrix infiltrated with immune cells (Figure 1G and Supplemental Figure 2B). The second harmonic visualization of NB and HGG confirmed that collagen deposition was absent in the former and present within the vasculature wall and tumor microenvironment of the latter (Supplemental Figure 2C). COL4A was expressed within the vasculature of NB, but COL1A and COL6A1/A2 were not expressed in NB by either second harmonic or multiplex imaging (Supplemental Figure 2C).

The expression of collagen receptors within the tumor microenvironment was explored (Figure 1E). LAIR1 expression was localized to the tumor and perivascular regions in HGG (Supplemental Figure 3A). The scRNA-Seq data demonstrated that *LAIR1* was expressed on myeloid and T cells (Figure 1E and Supplemental Figure 3, B and C). Immune expression of other collagen receptors was negligible. Multiplex imaging confirmed LAIR1 expression on myeloid cells expressing M2 markers within regions expressing COL6A (Figure 1H and Supplemental Figure 2D). Pan-cancer examination revealed a correlation between *COL6A1* and *LAIR1* expression and across glioma lineages (Supplemental Figure 3, D–F).

We had initially hypothesized that collagen might serve as a physical barrier to immune surveillance. Instead, we found CD11c^+^ immune cells embedded in collagen. LAIR1^+^ immune cells were found to be enriched in regions of collagen, indicating a potentially previously unappreciated mechanism of glioma-mediated immune suppression (Figure 1I). Currently, there is a phase I trial of a LAIR1 antagonist in myeloid malignancies (NextCure NCT05787496) that could be considered as a new treatment for collagen-enriched gliomas.

## Supplementary Material

Supplemental data

Supplemental video 1

Supporting data values

## Figures and Tables

**Figure 1:**
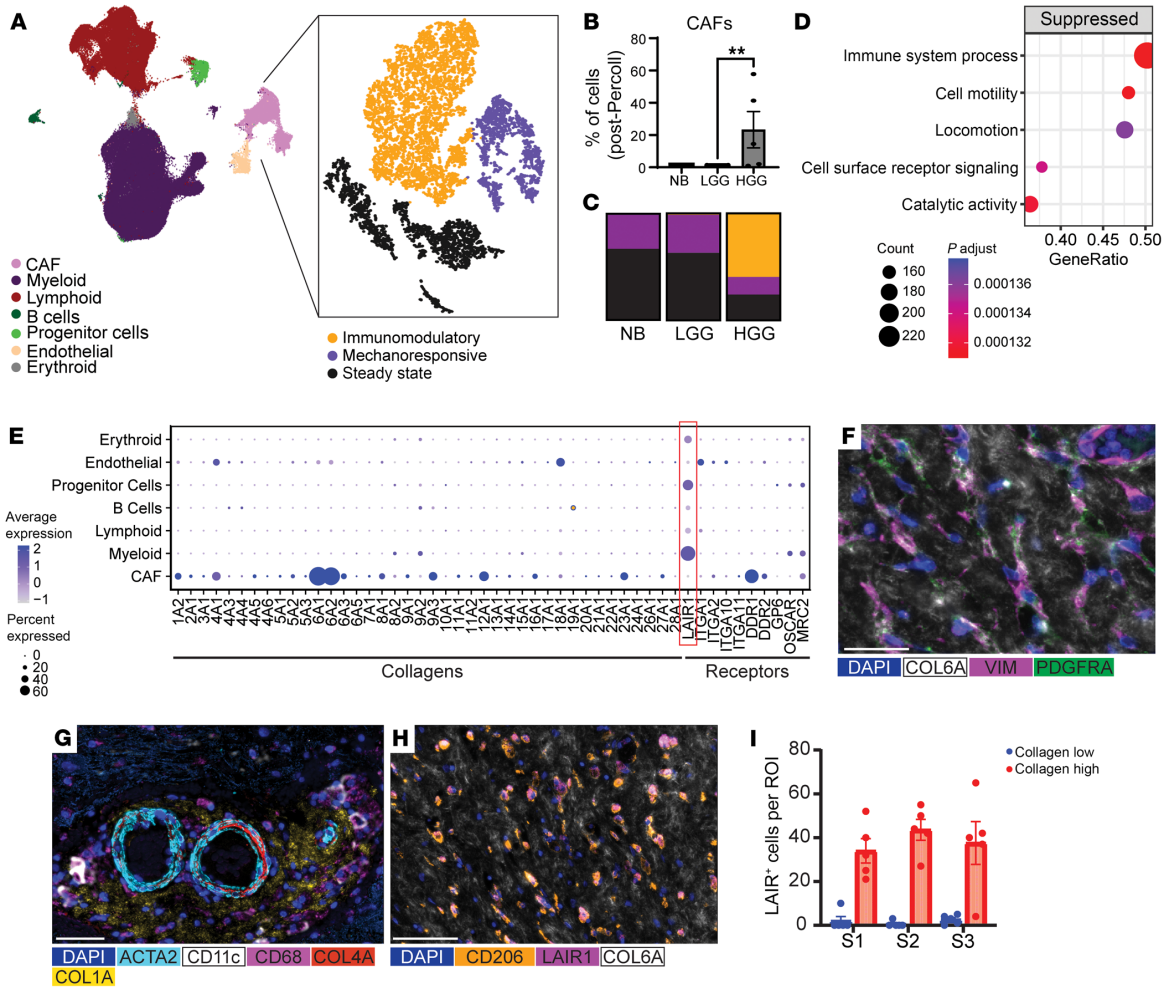
CAFs are enriched in pediatric HGG relative to LGG and NB. (**A**) UMAP clustering from 21 pediatric brain samples (13 PAs, 5 HGGs, and 3 NBs). (**B**) The frequency of CAFs is a function of glioma grade. Data represent mean ± SEM. **P < 0.01. LGG, low-grade glioma. (**C**) Stacked histogram of CAF subtypes by glioma grade. Color within the bar denotes the CAF subtype. (**D**) Bubble plot depicting gene ontology (GO) analysis of functions that are suppressed by CAFs. Each bubble represents a GO term, the bubble size corresponds to the gene ratio, and the color indicates the *P* value. (**E**) Dot plot displaying collagen and receptor expression within pediatric gliomas. Bubble size corresponds to the percentage of cells expressing gene marker; colors indicate average expression. (**F**) Multiplex imaging demonstrates PDGFRA^+^VIM^+^PDGFRB^+^ACTA2^+^ CAFs embedded in COL6A1/A2 (white) within HGG. Scale bar: 50 μm. See Supplemental Video 1. (**G**) ACTA2^+^COL4A^+^ arteries were embedded in a COL1A1/A2 matrix infiltrated with immune cells. Scale bar: 50 μm. (**H**) LAIR1^+^ myeloid cells embedded in COL6A1/A2^+^ areas. Scale bar: 50 μm. (**I**) Quantification of LAIR1^+^ cells in COL6A1/A2^+^ areas versus areas without COL6A1/A2 in HGG (*n* = 3 samples and 5 ROI per sample). Data represent mean ± SEM.

## Version 1 02/15/2024

Electronic publication
