# Supplementary material for: Cancer-associated fibroblast–secreted collagen is associated with immune inhibitor receptor LAIR1 in gliomas
Source: J Clin Invest. 2024 Feb 15;134(4):e176613. doi: 10.1172/JCI176613 (PMC10866651; doi:10.1172/JCI176613)
Supplement: Supplemental data [file jci-134-176613-s047.pdf]

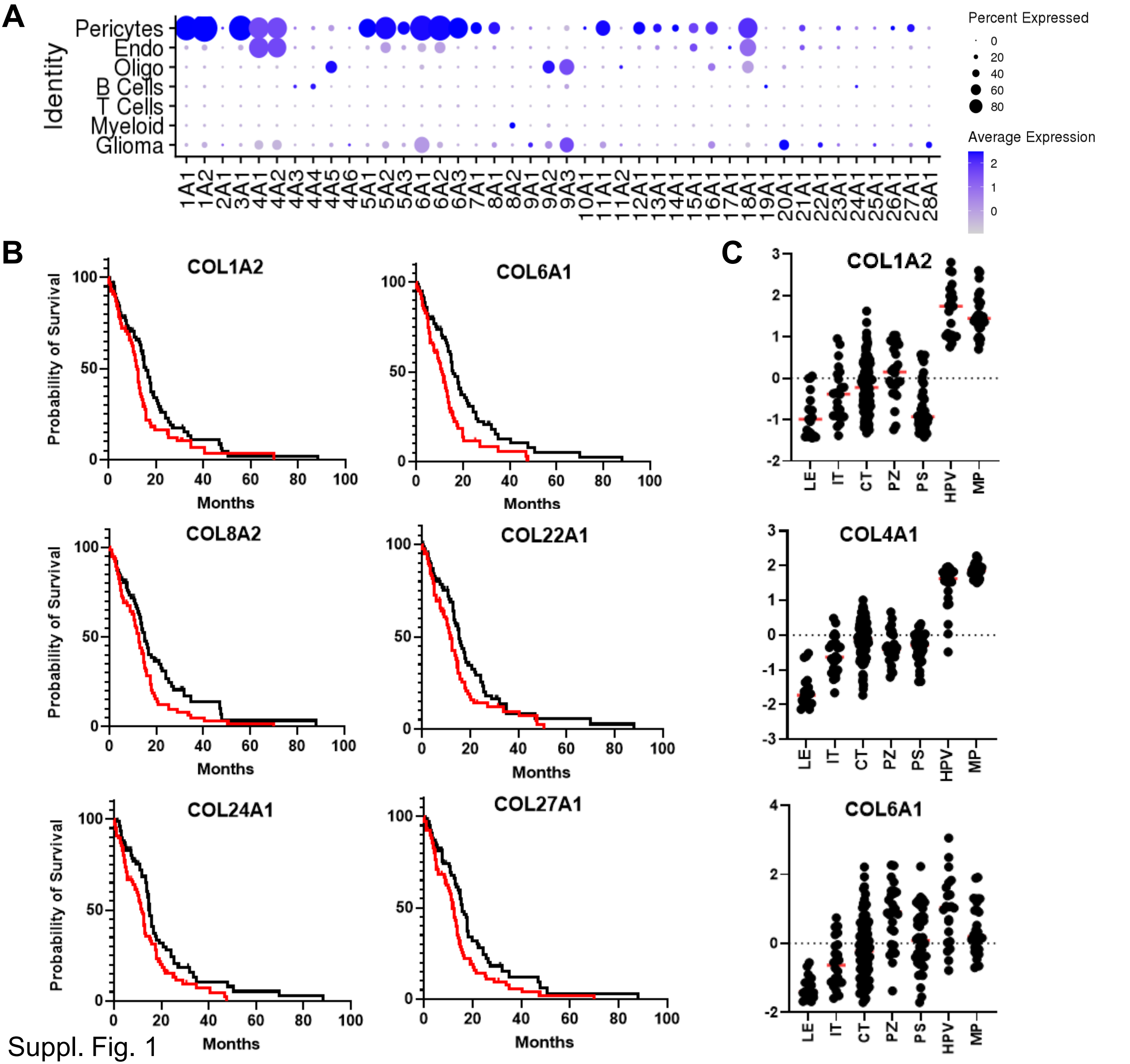

A
Identity
Pericytes
Endo
Oligo
B Cells
T Cells
Myeloid
Glioma
1A1
1A2
2A1
3A1
4A1
4A2
4A3
4A4
4A5
4A6
5A1
5A2
5A3
6A1
6A2
6A3
7A1
8A1
8A2
9A1
9A2
9A3
10A1
11A1
11A2
12A1
13A1
14A1
15A1
16A1
17A1
18A1
19A1
20A1
21A1
22A1
23A1
24A1
25A1
26A1
27A1
28A1
Percent Expressed
0
20
40
60
80
Average Expression
2
1
0
B
COL1A2
COL6A1
COL8A2
COL22A1
COL24A1
COL27A1
Probability of Survival
Months
0
50
100
0
20
40
60
80
100
C
COL1A2
COL4A1
COL6A1
LE
IT
CT
PZ
PS
HPV
MP

Suppl. Fig. 1

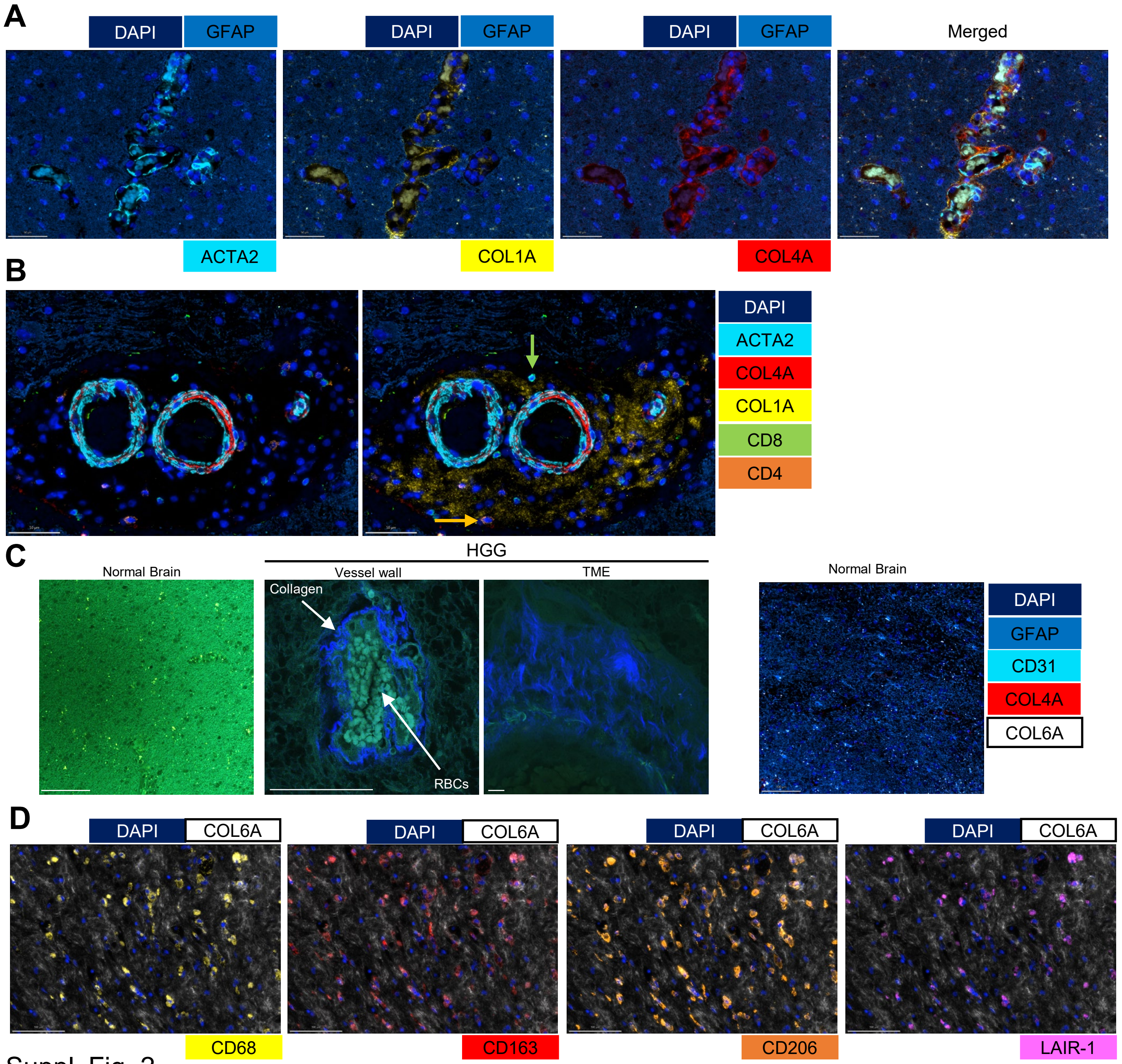
A
DAPI
GFAP
ACTA2
COL1A
COL4A
Merged
B
DAPI
ACTA2
COL4A
COL1A
CD8
CD4
C
Normal Brain
HGG
Vessel wall
TME
Collagen
RBCs
Normal Brain
DAPI
GFAP
CD31
COL4A
COL6A
D
DAPI
COL6A
CD68
CD163
CD206
LAIR-1

Suppl. Fig. 2

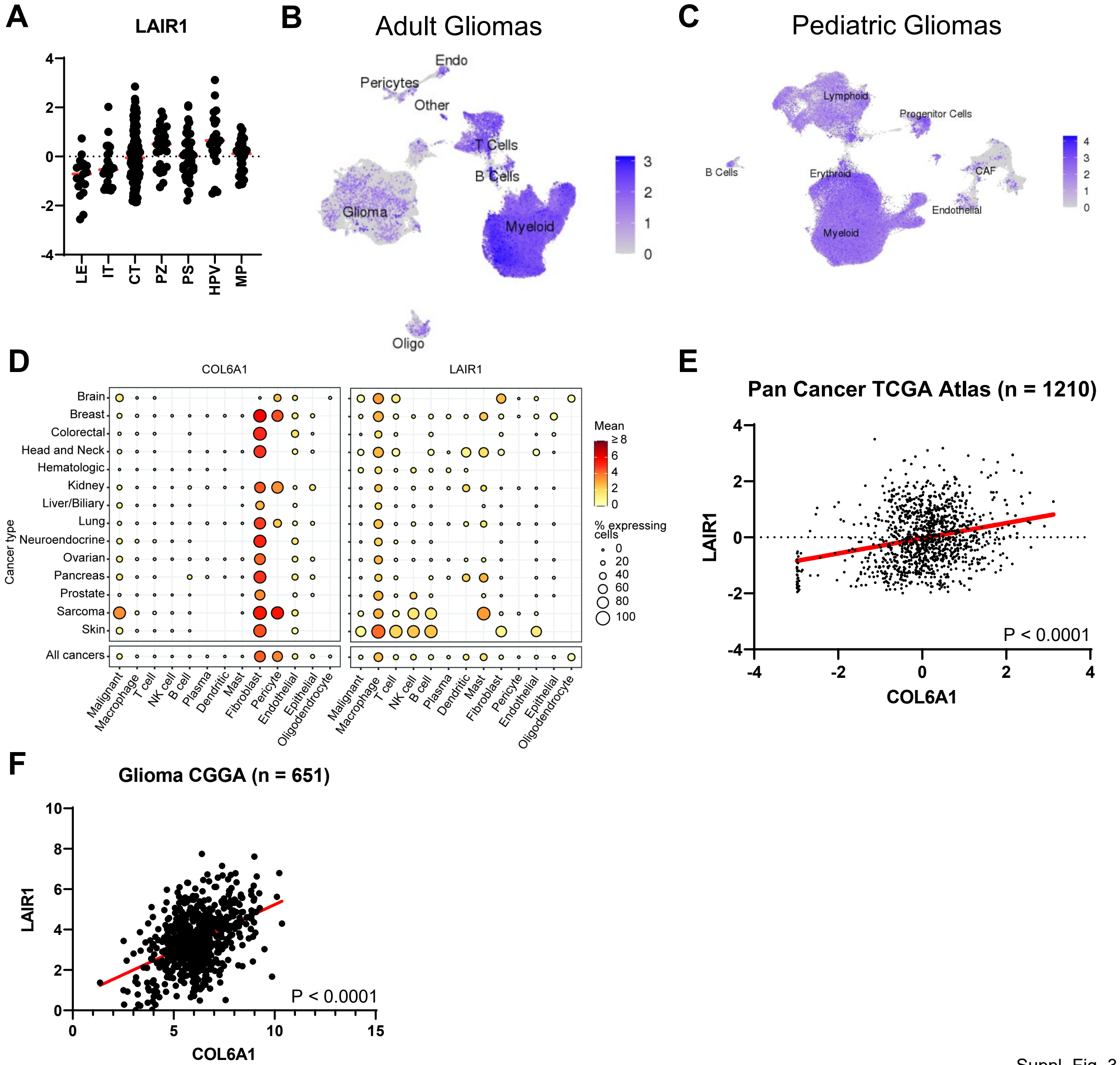
A
LAIR1
4
2
0
-2
-4
LE
IT
CT
PZ
PS
HPV
MP
B
Adult Gliomas
Endo
Pericytes
Other
T Cells
B Cells
Glioma
Myeloid
Oligo
3
2
1
0
C
Pediatric Gliomas
Lymphoid
Progenitor Cells
B Cells
Erythroid
CAF
Endothelial
Myeloid
4
3
2
1
0
D
COL6A1
LAIR1
Cancer type
Brain
Breast
Colorectal
Head and Neck
Hematologic
Kidney
Liver/Biliary
Lung
Neuroendocrine
Ovarian
Pancreas
Prostate
Sarcoma
Skin
All cancers
Malignant
Macrophage
T cell
NK cell
B cell
Plasma
Dendritic
Mast
Fibroblast
Pericyte
Endothelial
Epithelial
Oligodendrocyte
Mean
≥ 8
6
4
2
0
% expressing cells
0
20
40
60
80
100
E
Pan Cancer TCGA Atlas (n = 1210)
LAIR1
COL6A1
P < 0.0001
4
2
0
-2
-4
F
Glioma CGGA (n = 651)
LAIR1
COL6A1
P < 0.0001
10
8
6
4
2
0
5
15

Suppl. Fig. 3

Supplementary Figure Legends

Suppl. Fig. 1. mRNA expression of top Fibroblast DEGs in the TCGA dataset. A) Collagen expression in cell populations present within the GBM TME. Pericytes, which also include fibroblasts, were the main source of type 1, 3, 4, 5, 6, and 18 collagens with the endothelial cell contributing to types 4 and 18. Subsets of oligodendrocytes produced types 4 and 9 and glioma cells produced types 4, 6, and 9. B cells, T cells, and myeloid cells did not produce significant amounts of collagen. Bubble size corresponds to the percent of cells expressing gene marker; colors indicate average expression. **B**) Six collagen subtypes are associated with a negative prognostic outcome in IDH-WT glioblastoma: *COL1A2* (high versus low expression: 12.3 vs 16.1 months; HR=0.68; p=0.03); *COL6A1* (high versus low expression: 11.9 vs 15.9 months; HR=0.53; p=<0.01); *COL8A2* (high versus low expression: 12.6 vs 15 months; HR=0.65; p=0.02); *COL22A1* (high versus low expression: 11.8 vs 15.4 months; HR=0.67; p=0.03); *COL24A1* (high versus low expression: 11.8 vs 15 months; HR=0.64; p=0.01); and *COL27A1* (high versus low expression: 11.8 vs 15.8 months; HR=0.63; p=0.01). **C**) RNA sequencing data from the Ivy Glioblastoma Atlas project was analyzed based on differences in the anatomical locations of the most frequently expressed collagens: *COL1A2, COL4A1*, and *COL6A1*. Z-score normalized mRNA expression is plotted. LE, leading edge; IT, infiltrating tumor; CT, cellular tumor; PZ, perinecrotic zone; PS, pseudopalisading cells around necrosis; HPV, hyperplastic blood vessels in cellular tumor; MP, microvascular proliferation.

Suppl. Fig. 2. A) Sequential multiplex imaging demonstrating, from left to right, ACTA2 (cyan blue), COL1A1/A2 (yellow), and COL4A (red) expression in HGG glomeruloid structures. Scale bar: 50 µm. **B**) Sequential multiplex imaging demonstrating CD8 (green) and CD4 (yellow) T cells embedded in COL1A1/A2 perivascular matrix with ACTA2+COL4A+ vessels. Arrows denote T cells which are almost exclusively in the perivascular regions. Scale bar: 50 µm. These images correspond to Fig. 1G. **C**) Second harmonic generation (SHG) showing collagen expression (in blue) in the vessel wall and the TME of HGG, and its absence in a control normal brain. Eight separate fields were imaged for each sample, and two individual patient samples were imaged per tumor type. Additionally, parallel multiplex imaging of control normal brain showing absence of collagen. Scale bars: 100 µm. **D**) Sequential multiplex imaging demonstrating, from left to right, CD68+ (yellow), CD163+ (red), CD206+ (orange) macrophages expressing LAIR1 (purple) within COL6A (white) enriched areas in HGG. Scale bar: 100 µm. These images correspond to Fig. 1H.

Suppl. Fig. 3. Spatial and cellular localization of LAIR1 expression in public datasets. **A**) Ivy-Atlas database analysis of LAIR1 expression. Z-score normalized mRNA expression is plotted. CT, cellular tumor; HPV, hyperplastic blood vessels in cellular tumour; IT, infiltrating tumour; LE, leading edge; MP, microvascular proliferation; PS, pseudopalisading cells around necrosis; PZ, perinecrotic zone. **B**) Feature plot showing *LAIR1* expression within adult gliomas; color indicates average expression. Data is from scRNA-seq of 44 tumor fragments representing 18 glioma patients (2 LGG, 11 newly diagnosed (nd) GBM, and 5 recurrent (r) GBM) from [18]. **C**) Feature plot showing *LAIR1* expression within our pediatric glioma cohort; color indicates average expression. **D**) Plot showing the average expression level of *COL6A1* and *LAIR1* and the percentage of cells expressing *COL6A1/LAIR1* in each cancer type and each of the most common cell types, namely those cell types constituting at least 1% of at least 5 individual datasets. Expression levels are defined as log2(TPM/10). Average expression levels and percentages were measured per cell type within each dataset, then averaged across studies within each cancer type. Obtained from the Curated Cancer Cell Atlas which comprised 71 studies accounting for 1375 samples and 2,310,177 cells. **E**) Scatter plot of *COL6A1* and *LAIR1* expression within the TCGA Pan Cancer Atlas (n=1210, F-Test: P-value <0.0001; R^2= 0.07). The mRNA expression z-scores (log FPKM capture) are displayed. **F**) Scatter plot of *COL6A1* and LAIR1 expression within the Chinese Glioma Genome Atlas (CGGA) (n=651, F-Test: P-value <0.0001; R^2= 0.20). The log2 mRNA expression values are displayed.

Video 1: Sequential multiplex imaging demonstrates that VIM+ACTA2+PDGFRB+PDGFRA+ CAFs were embedded in COL6A1/A2 within high-grade gliomas. COL6A1/A2 is also identified within the cytoplasm of the CAFs.

SUPPLEMENTARY METHODS

Study approval. Under STU00214485, approved by the institutional review board of Northwestern University, and 2021-4677, approved by the Ann and Robert H. Lurie Children's Hospital of Chicago, patients were identified with surgically resectable tumors. All patients were screened based on a presumptive radiographic diagnosis of central nervous system (CNS) glioma or brain metastasis. Written informed consent was obtained from all participants or their guardians.

Tissue Processing and Preparation. Patient tumors were graded pathologically by the study neuropathologists (CMH, NW) according to the World Health Organization classification (1). At least 500 mg of the viable tumor was required to obtain enough for analysis and was processed within 30 min-1hour after resection. The normal brain was obtained from subjects as part of the planned surgical approach to gain access to a low-grade non-infiltrating glioma or during a planned super-total resection of adjacent regions that lacked T2/Flair abnormality on MRI imaging. The normal brain was sent for analysis separately from the tumor. The freshly resected tissue was processed in parallel both for a single cell suspension and formalin-fixed paraffin embedded (FFPE) analysis. For the former, the tissue was minced into small pieces using a scalpel, dissociated, and suspended using a Pasteur pipette in 10ml Iscove's DMEM (IMDM; Iscove's Modification of DMEM) 1X (Corning) containing 2% inactivated Fetal Bovine Serum (FBS; Sigma Aldrich) and Collagenase and DNase enzymes at final concentrations of 100µg/ml and 20units/ml, respectively. The prepared mixture was incubated for 35-40mins at 37°C with agitation. After tissue filtration using a 70-µm nylon cell strainer (BD Biosciences) and a brief centrifugation at 4°C, the pellet was resuspended in 20ml mix of 5.4ml of Percoll™ Plus (GE Healthcare) overlaid with 12ml of 1X Phosphate Buffered Saline (PBS) and 0.6ml of 10X PBS (Corning). The tube was centrifuged at 800*g* for 10min at 4°C, with 9 acceleration and 0 deceleration. After centrifugation, the immune enriched cell pellet was collected, washed, stained with Trypan blue dye (Sigma-Aldrich), and counted using Countess II FL automated cell counter in a Countess cell counting chamber (Invitrogen). The FFPE slides were collected from the Neurological Surgery Tumor Bank (NSTB) of Northwestern University. Slides of tissue of 4 µm thickness were prepared, mounted on positively charged glass slides (Super Frost Plus microscope slides, ThermoFisher), and stored at room temperature for subsequent staining analysis. For each case, 1 H&E slide was reviewed, and tissue was segmented by a certified neuropathologist (CMH).

Human CAF cell line. A human CAF were obtained from Dr Manish Aghi as described in Jain et.al 2021 (2) and grown in DMEM/F-12 plus 10% FBS and 1% penicillin/streptomycin. To measure elaborated collagen, the hydroxyproline assay kit (Chondrex 6017) was utilized.

Single-cell RNA-Sequencing. Single-cell sequencing was carried out using the chromium Next GEM Single Cell protocol (10x genomics). Post-library preparation cells were sequenced using the Illumina Novaseq. Raw data was preprocessed and aligned using Cell Ranger to obtain the matrix and count files. Seurat R Package using scRNA-seq Seurat10x genomic workflow was then used for all subsequent analyses unless noted otherwise. After filtering using a percent mitochondrial DNA threshold of 20% and UMI range of 200 to 15000, 186,317 cells were included for further analysis. Cells were then subject to Log Normalize, Scale Data, and PCA functions. Find Clusters and Find Markers functions were utilized for clustering and marker identification and non-linear dimensional reduction techniques were applied to visual data in UMAP plot format Cell clusters were annotated using three methods to produce robust cell assignments: 1) comparison against known cell markers; 2) examination of DEGs with the Human Protein Atlas; and 3) ScType R package, an automated cell assignment algorithm was utilized. The following markers were used for CAFs: *ACTA2, FAP, PDGFRA, PDGFRB, VIM, PDPN, S100A4, TNC*, and *COL1A1*. Additionally, subtypes of CAFs were defined based on expression of immunomodulatory - *CXCL12, DCN, STAT1 and SPON2*; mechanoresponsive – *COL6A1, POSTN, COL4A1, PDGFRB, MGP*, and *FOSB*; and steady-state - *FAP* and *TIMP2*. DEGs were used for Gene Ontology enrichment analysis using the Bioconductor Package Cluster Profiler (3). Significantly enriched GO-BP (Gene Ontology-Biological processes) terms were retrieved by setting the threshold of FDR=3; queried genes were manually selected using immunological keywords. Results were displayed using bubble plots. Each bubble represents a GO term, the bubble size corresponds to the gene ratio and the color indicates the P-value.

Automated Hyperplex Immunofluorescence Staining and Imaging on the COMET™ system. In brief, FFPE slides were preprocessed for antigen retrieval using the PT Module (Epredia) with Dewax and HIER Buffer H (TA999-DHBH, Epredia) for 60 minutes at 102°C. Subsequently, slides were rinsed and stored in a Multistaining Buffer (BU06, Lunaphore Technologies) till use. The 20-plex protocol template was generated using the COMET™ Control Software, and reagents were loaded onto the device to perform the sequential immunofluorescence (seqIF™) protocol. CAFs express fibroblast-associated markers however no single marker is shared by all CAFs. The most frequently expressed and used marker is ACTA2 (α-smooth muscle actin) (4). The latter has been

described as being expressed in CAFs in a glioma setting (5). The antibodies used for this analysis were: CD31 (endothelial cells, Abcam, cat#Ab225883, clone EPR17259), GFAP (glioma tumor cells, Abcam, cat#Ab58428, clone EPR1034Y), SOX2 (stem cells, Abcam, cat#Ab92494, clone EPR3131), PDGFRB (Cell signaling, cat#3169, clone 28E1), ACTA2 (Abcam, cat#Ab7517, clone 1A4), PDGFRA (Cell signaling, cat#3174, clone D1E1E XP), and Vimentin (VIM, Cell signaling, cat#5741, clone D21H3 XP) (CAFs), COL1A1/2 (Abcam, cat#Ab138492, clone EPR7785, COL4A1/2 (Abcam, cat#ab6586, polyclonal), COL6A1/2 (Abcam, cat#Ab182744, clone EPR17072) and COL6A3 (Sigma, cat#HPA010080, polyclonal) (collagen types), CD4 (helper T cells, Abcam, cat#Ab133616, clone EPR6855), CD8 (cytotoxic T cells, Leica, cat#PA0183, clone 4B11), P2RY12 (microglia, Atlas antibodies, cat#HPA014518, polyclonal), CD68 (pan-monocyte/macrophage marker, Dako Agilent, cat#M0876, clone PG-M1), CD11c (antigen-presenting cells, Abcam, cat#Ab52632, clone EP1347Y), CD163 (macrophage scavenger receptor, Abcam, cat#Ab182422, clone EPR19518), CD205 (dendritic cells, Abcam, cat#Ab124897, clone EPR5233), CD206 (immune suppressive macrophages, Abcam, cat#ab64693, polyclonal), and LAIR-1 (collagen binding receptor, Santa Cruz, cat#sc-398141, clone F-5). The secondary antibodies used were Alexa Fluor Plus 647 goat anti-rabbit (Thermo Scientific, cat#A32733, 1/400 dilution) and Alexa Fluor Plus 555 goat anti-mouse (Thermo Scientific, cat#A32727, 1/200 dilution). The nuclear signal was detected with 4',6-diamidino-2-pheynlindole (DAPI) (Thermo Scientific) by dynamic incubation of 2min. All antibodies were diluted in a Multistaining Buffer. For each cycle the following exposure times were used: DAPI 80ms, TRITC 2min, Cy5 2min, and primary antibody 4min. The elution step lasted 2 minutes for each cycle and was performed with Elution Buffer (BU07-L, Lunaphore Technologies) at 37°C. The quenching step lasted for 30 seconds and was performed with a Quenching Buffer (BU08-L, Lunaphore Technologies). The imaging step was performed with Imaging Buffer (BU09, Lunaphore Technologies). The seqIF™ protocol in COMET™ resulted in a multi-stack ome.tiff file where the imaging outputs from each cycle are stitched and aligned. COMET™ ome.tiff contains a DAPI image, intrinsic tissue autofluorescence in TRITC and Cy5 channels, and a single fluorescent layer per marker.

Spatial Bioinformatic analysis of multiplex images. The multiplex files generated were then analyzed using the Viewer™ (Lunaphore Technologies) where markers were pseudo-colored, and no background subtraction was applied in this setting. Subsequent bioinformatic analysis for LAIR-1 quantification using the Visiopharm® software (version January 2023) was conducted. Multiple regions of interest (n=10 total; n=5 high COL6A regions vs n=5 COL6A low regions) were

collected from each of the samples and LARI-1+ cells were counted and compared between areas.

Second harmonic generation (SHG) imaging. SHG imaging was used to investigate the organized collagen structures in human GBM samples. The first 10-micron FFPE samples were deparaffinized, hydrated, and kept in DI water until imaging. Multiphoton imaging was performed on a Nikon A1R-MP upright microscope equipped with transmitted light detectors and a Nikon A1-NDN water immersion condenser. Images were captured with a 25X water immersion objective (Apo LWD 25x 1.10W DIC N2) and a Chameleon pulsed laser (Coherent) tuned to 820 nm. z-stacks were taken for each image (5um steps, 3 steps total). The acquired data was processed through background subtraction and spatial filtering for noise reduction. Data was analyzed using Nikon Elements software (you might need more details on how you did the quantification) All experiments were performed at room temperature, and image acquisition settings were kept consistent throughout the study to ensure comparability between samples and replicates. Eight separate fields were imaged for each sample, and two individual patient samples were imaged per tumor type.

Online datasets analysis. A cohort of 156 patients with HGG from the TCGA dataset and 651 from the CGGA was obtained from GlioVis (gliovis.bioinfo.cnio.es). Kaplan-Meir curves using Log2-transformed mRNA expression of selected markers (*COL1A2, COL6A1, COL8A2, COL22A1, COL24A1, COL27A1*) were downloaded and analyzed using Gliovis inbuilt analysis tools. Spatial RNA-Seq data were collected from the Ivy Glioblastoma Atlas Project (IVY-GAP). IVY-GAP contained 41 patients whose tumor samples were classified based on anatomic features. Z-score normalization of RNA-Seq expression of *COL1A2, COL4A1*, and *COL6A1* were downloaded and visualized using scatter plots. A publicly available scRNA database from Abdelfattah et. al. was utilized using the online platform through the Broad Institute to create dot plots of collagen expression in adult gliomas (6). Additionally, for pan-cancer analysis, cBioPortal was utilized to query the ICGC/TCGA for *COL6A1* and *LAIR1* mRNA expression values.

Statistics. All statistical analyses were performed using GraphPad Prism software version 9.4.0. Survival analysis between control and experimental groups was determined by the Kaplan-Meier method, and statistical significance was assessed using the log-rank test (Mantel-Cox). Multiple comparisons were not performed, and no samples were excluded from analysis. The p values for

curve comparisons were calculated using the Log-rank method followed by Bonferroni correction. The following were considered statistically significant: **p<0.01; n.s. not significant.

Data Availability.
TCGA data is available from GlioVis (gliovis.bioinfo.cnio.es). Ivy Glioblastoma Atlas Project data is available from IVY-GAP (https://glioblastoma.alleninstitute.org/rnaseq/search/index.html). Sequencing data has been deposited in the National Center for Biotechnology Information Gene Expression Omnibus (GEO GSE249263).

Author Contributions. ABH provided experimental design and/or implementation. ST, HN, CMH, NW, SL, and MD collected the data. ST, HN, MD, and ABH analyzed the data. ST and ABH wrote the manuscript. All authors read and approved the final version of the manuscript.

Acknowledgments. This research was supported by NIH grants CA120813, NS120547, CA221747, and CA060553, institutional funding from the Lou and Jean Malnati Brain Tumor Institute of the Lurie Comprehensive Cancer Center, and gifts from the Mosky family and the Stephen Coffman Trust. Special thanks to Marie Belle Najem for her expertise in animated video generation.

Conflict of Interest: ABH serves on the advisory board of Caris Life Sciences and the WCG Oncology Advisory Board; receives royalty and milestone payments from DNAtrix for the licensing of the patent "Biomarkers and combination therapies using oncolytic virus and immunomodulation" (no. 11,065,285); is supported by research grants from Celularity, Alnylam, and AbbVie, and receives consulting fees from Novocure and Istari Oncology. She additionally has active granted patents titled "miRNA for treating cancer and for use with adoptive immunotherapies" (no. 9,675,633) and "Concurrent chemotherapy and immunotherapy" (no. 9,399,662), with a patent pending, "Low intensity ultrasound combination cancer therapies" (international applications PCT/US2022/019435 and US 63/158,642). The remaining authors declare no potential conflict of interest.

1. Louis DN, Perry A, Wesseling P, Brat DJ, Cree IA, Figarella-Branger D, et al. The 2021 WHO Classification of Tumors of the Central Nervous System: a summary. Neuro Oncol.

2021;23(8):1231-51. Epub 2021/06/30. doi: 10.1093/neuonc/noab106. PubMed PMID: 34185076; PubMed Central PMCID: PMCPMC8328013.
2. Jain S, Rick JW, Joshi R, Beniwal A, Spatz J, Chang AC-C, et al. Identification of Cancer-Associated Fibroblasts in Glioblastoma and Defining Their Pro-tumoral Effects. bioRxiv. 2021:2021.05.08.443250. doi: 10.1101/2021.05.08.443250.
3. Yu G, Wang LG, Han Y, He QY. clusterProfiler: an R package for comparing biological themes among gene clusters. OMICS. 2012;16(5):284-7. Epub 2012/03/30. doi: 10.1089/omi.2011.0118. PubMed PMID: 22455463; PubMed Central PMCID: PMCPMC3339379.
4. Nurmik M, Ullmann P, Rodriguez F, Haan S, Letellier E. In search of definitions: Cancer-associated fibroblasts and their markers. Int J Cancer. 2020;146(4):895-905. Epub 20190228. doi: 10.1002/ijc.32193. PubMed PMID: 30734283; PubMed Central PMCID: PMCPMC6972582.
5. Trylcova J, Busek P, Smetana K, Balaziova E, Dvorankova B, Mifkova A, et al. Effect of cancer-associated fibroblasts on the migration of glioma cells in vitro. Tumor Biology. 2015;36(8):5873-9. doi: 10.1007/s13277-015-3259-8.
6. Abdelfattah N, Kumar P, Wang C, Leu JS, Flynn WF, Gao R, et al. Single-cell analysis of human glioma and immune cells identifies S100A4 as an immunotherapy target. Nat Commun. 2022;13(1):767. Epub 2022/02/11. doi: 10.1038/s41467-022-28372-y. PubMed PMID: 35140215; PubMed Central PMCID: PMCPMC8828877 interests.
